# Barriers and facilitators when implementing midwifery continuity of carer: a narrative analysis of the international literature

**DOI:** 10.1186/s12884-024-06649-y

**Published:** 2024-08-14

**Authors:** Aimee Louise Middlemiss, Susan Channon, Julia Sanders, Sara Kenyon, Rebecca Milton, Tina Prendeville, Susan Barry, Heather Strange, Aled Jones

**Affiliations:** 1 School of Nursing and Midwifery, University of Plymouth, Plymouth, UK; 2 Centre for Trials Research, Cardiff University, Cardiff, UK; 3 School of Healthcare Sciences, Cardiff University, Cardiff, UK; 4 Institute of Applied Health Research, University of Birmingham, Birmingham, UK; 5 Women’s Health Research Centre, Imperial College London & Imperial College NHS Trust, London, UK; 6 Division of Women’s Children’s and Clinical Support, Imperial College Healthcare NHS Trust, London, UK

**Keywords:** Midwifery, Midwifery continuity of carer, Maternity care, Implementation, CFIR, Policy

## Abstract

**Background:**

Midwifery continuity of carer (MCoC) is a model of care in which the same midwife or small team of midwives supports women throughout pregnancy, birth and the postnatal period. The model has been prioritised by policy makers in a number of high-income countries, but widespread implementation and sustainability has proved challenging.

**Methods:**

In this narrative review and synthesis of the global literature on the implementation and sustainability of midwifery continuity of carer, we identify barriers to, and facilitators of, this model of delivering maternity care. By mapping existing research evidence onto the Consolidated Framework for Implementation Research (CFIR), we identify factors for organisations to consider when planning and implementing midwifery continuity of carer as well as gaps in the current research evidence.

**Results:**

Analysing international evidence using the CFIR shows that evidence around midwifery continuity of carer implementation is patchy and fragmented, and that the impetus for change is not critically examined. Existing literature pays insufficient attention to core aspects of the innovation such as the centrality of on call working arrangements and alignment with the professional values of midwifery. There is also limited attention to the political and structural contexts into which midwifery continuity of carer is introduced.

**Conclusions:**

By synthesizing international research evidence with the CFIR, we identify factors for organisations to consider when planning and implementing midwifery continuity of carer. We also call for more systematic and contextual evidence to aid understanding of the implementation or non-implementation of midwifery continuity of carer. Existing evidence should be critically evaluated and used more cautiously in support of claims about the model of care and its implementation, especially when implementation is occurring in different settings and contexts to the research being cited.

**Supplementary Information:**

The online version contains supplementary material available at 10.1186/s12884-024-06649-y.

## Background

Midwifery continuity of carer (MCoC) is a model of maternity care organisation which aims for a woman and her baby to be cared for by the same midwife, or small team of midwives, throughout pregnancy and birth, including the antenatal, intrapartum and postnatal periods [1]. It contrasts with models of maternity care where staffing is organised by care location, such as primary care or hospital, and care across the different stages of pregnancy and birth is provided by staff who work shift patterns in different clinical locations including community settings, hospital delivery suite, or hospital postnatal wards. Under such models women can expect to receive care from numerous different midwives. Models of maternity care prioritising continuity form part of a general drive in many healthcare systems towards ‘continuity of care’ as a way of caring for individuals over time through informational, management, relational or interpersonal, and geographic continuity [2, 3]. However, the model of continuity which is prioritised in maternity service provision is a specific form which prioritises the relational or interpersonal aspects of continuity by emphasizing ‘continuity of carer’. In this form, the same individual should carry out all, or the majority of, the care. This is distinct from other continuity models in healthcare where ‘continuity of care’ is achieved through providing the same type or standard of care across multiple individuals in a team. Furthermore, in midwifery continuity of carer models the individual carer in question is a midwife rather than, for example, a General Practitioner or obstetrician. Continuity of midwifery carer aligns with the midwifery professional ethos of relational ‘with woman’ care and with an ongoing negotiation in which midwifery as a profession has sought to establish its role in pregnancy and birth in relation to care provided by doctors [4, 5].

Achieving continuity of carer through the presence of the same midwife at all antenatal and postpartum care encounters, and during labour and birth, has complex workforce deployment consequences. Options include one-to-one caseload-style midwifery where one named midwife provides all care for specific women; a buddy system where two midwives provide backup for one another for leave, off-call or sickness; and team midwifery models where groups of various sizes support one another, ideally with women meeting the whole team before labour. Policy sometimes conflates having a ‘known midwife’ at birth, achievable through the buddy system or team midwifery, with full continuity of all care by one person [6]. Which of these staffing systems achieves the highest levels of MCoC is not established in the literature. For example, team midwifery has been found to result in the most intrapartum continuity, but as team size increased intrapartum continuity by a specific carer decreased [7], and larger teams also raise the question of whether meaningful relationships between women and multiple midwives are established [8]. However, smaller teams reduce the chance of intrapartum continuity where limits to working hours apply. Continuity across the three phases of care may also be prioritised at the expense of continuity within phases [9]. There is potentially a trade-off between the closeness of the relationship between a woman and a single midwife, and the presence of a ‘known’ midwife at birth. In a team midwifery system where a woman is cared for antenatally and postnatally by a larger group of midwives, one of whom may be present during labour and birth, it is possible that the mechanisms which are thought to provide the clinical benefits of continuity may not be activated.

Despite these complexities there has been a strong drive towards the implementation of MCoC in several high-income healthcare systems such as Australia, Scotland, and England. An influential systematic review reported that MCoC results in several improved clinical outcomes for women and babies in midwife-led continuity of care, including lower likelihood of instrumental vaginal birth, lower use of regional anaesthesia, and decreased preterm birth and all fetal loss and neonatal death [10]. The review was widely cited as the rationale for implementing midwifery continuity of carer, although it has been critiqued for not accounting for differences in health service settings and contexts [11]. The model is also repeatedly represented as a more satisfying way of working for midwives (see for example [12–14]). MCoC is supported by international institutions such as the World Health Organisation (WHO) [15] and the International Confederation of Midwives (ICM) [16]. Internationally, it is the standard model of care in New Zealand [17], is provided to many women in Denmark [5], and is a policy priority in Australia [18] where provision has reached around 8% of women [19]. In England, a policy emphasis on continuity in maternity care dates back to 1993 and the *Changing Childbirth* report led by Baroness Cumberlege [20]. The model was then given renewed impetus with Baroness Cumberlege’s second review of maternity care in 2016, *Better Births*, [21] which was integrated into long term National Health Service (NHS) and government strategy in England [22, 23]. MCoC has also been included in recent maternity care policy in Scotland and Northern Ireland [6, 24]. However, the model of care has been critiqued in England in the context of difficulties with overall midwifery staffing levels [25] and the roll out of the policy is currently limited to where safe midwifery staffing levels can be evidenced [26]. The MCoC implementation and sustainability difficulties currently experienced in England have also been noted in the past in England and Wales [27], and in Australia [28].

The implementation of MCoC in maternity care is thus a priority for policy makers in several settings but limited attention has been paid to research identifying the broad barriers and facilitators of the model’s adoption. Reviews of the literature concerned with MCoC implementation to date tend to have a specific scope and focus, including assessing feasibility in specific contexts such as rural Australia [29], scoping where MCoC takes place globally [30], reviewing the cost effectiveness of MCoC during complex pregnancies as a possible facilitator of implementation [31], considering the role of leadership and management in effecting MCoC implementation [32], and describing midwives’ perceptions of barriers and enablers to working in MCoC [28, 33, 34]. Other reviews make limited implementation claims in the course of a discussion of general aspects of MCoC research [35, 36]. One previous review addresses MCoC implementation processes more widely using Normalisation Process Theory (NPT) [37]. It sought to understand the process of MCoC implementation itself through NPT’s focus on the work which is done, finding that key factors are the willingness of midwives to work in the model and the provision of ‘organisational space’ in the NHS in the United Kingdom (UK).

Our narrative synthesis [38] complements this work by critically applying the Consolidated Implementation Framework (CFIR) [39, 40] in order to understand the complexity of MCoC implementation and identify gaps in the existing research literature in terms of barriers and facilitators of implementation. The analytic focus on factors such as the innovation itself and the multiple overlapping contexts within which it is introduced is especially useful in understanding barriers to and facilitators of MCoC implementation and other complex interventions in maternity and other healthcare services.

## Methods

**Fig. 1:**
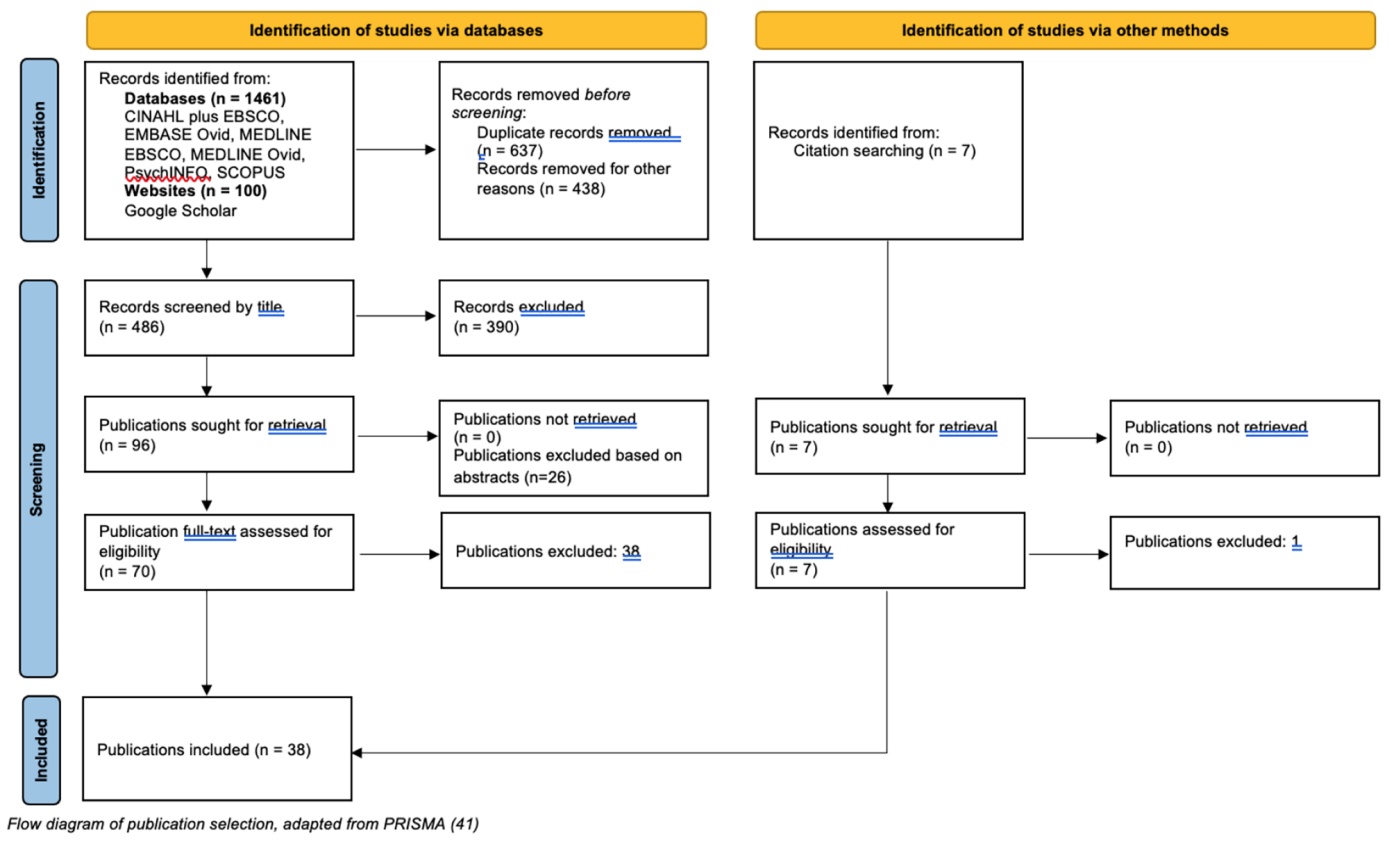
Flow diagram of publication selection

The aim of our study was to review international literature relating to the implementation of midwifery continuity of carer in order to identify barriers and facilitators of this process and to analyse the scope for future research in this area. We selected a narrative synthesis as appropriate way to appraise mixed methods and qualitative studies through an interpretive and critical lens [38]. We carried out a literature search in July 2023 using databases, internet searches, and citation lists from published literature and literature reviews on MCoC. Search terms and inclusion and exclusion criteria for the review were determined by the authors with the advice of an information specialist after a separate scoping search of literature reviews in the field of MCoC which allowed us to identify keywords, synonyms, and terminological variations. We conducted database searches using free text and database-specific MeSH subject headings (‘Midwifery’ and ‘continuity of care*’) and combined these via AND with ‘implement* OR barrier* OR enabl* OR facilitat*’. We conducted searches for literature from 1993 onwards, when MCoC first became a policy priority in England, and selected databases on the basis of our scoping analysis of previous literature reviews, as well as the advice of the information specialist, and the team’s experience of undertaking similar reviews. Databases searched were CINAHL Plus EBSCO, EMBASE Ovid, MEDLINE EBSCO, MEDLINE Ovid, PsychINFO, and SCOPUS. We also conducted a search on Google Scholar and screened the first 100 results. The selection of included publications is represented in the attached flow diagram adapted from PRISMA [41] (Fig. 1), which was carried out by two team members. We excluded other literature reviews, research related to the clinical effectiveness of MCoC rather than implementation of the model or factors which could affect implementation, research not related to maternity services or midwifery, research focused on midwifery students, articles not reporting primary empirical research (such as editorials, opinion and news articles) and articles focused on other topics where continuity of carer was not the main focus (for example, breastfeeding outcomes).

Of the 38 papers reviewed, 18 presented evidence from Australia, 13 from the UK (Scotland and England), four from New Zealand and three from other settings (Denmark, Canada). Of the 13 research papers from the UK, eight were published since 2016 when the policy context reprioritised the delivery of MCoC, with the other UK research based on the post-1993 policy context. Further detail regarding the selected papers is presented in Additional File 1.

We thematically analysed the findings of the selected publications [42, 43] to inductively draw out descriptive factors representing barriers or facilitators of MCoC implementation or sustainability. The purpose of coding as inductively as possible in the first stage was to avoid imposing an analytic or theoretical framework too early in the process in case this rendered some data invisible. The coding could not be completely inductive, however, since we were interested in identifying barriers and facilitators to implementation rather than all aspects of MCoC. Our process was a balance between theoretical and inductive coding, producing data in which codes were initially identified, then related to one another in a gathering and mapping exercise to iteratively build thematic connections. Connections and findings were regularly discussed with all authors to explore and clarify the ideas as part of this inductive process. We then mapped our themes onto the updated Consolidated Framework for Implementation Research (CFIR) [39, 40] to aid analysis of factors affecting the implementation of MCoC and identify gaps in the MCoC implementation literature. An example of this process moving from raw data to the CFIR constructs is given in Additional File 2. The CFIR is a widely used analytic framework in implementation science which identifies contextual factors which affect implementation in the real world [39]. The CFIR consists of five domains, further subdivided into constructs, which consider implementation factors related to the nature of the innovation itself, the outer and inner settings in which it is implemented, the individuals who implement the innovation, and the implementation process (Table 1). Our results follow this framework and are presented under subheadings related to each of the CFIR constructs.

**Table 1:** The CFIR constructs and definitions from Damschroder et al. 2022

CFIR construct	Definition
Innovation domain	The “thing” being implemented, in this case a MCoC model of care
Outer setting domain	The setting in which the Inner Setting exists, e.g., hospital system, district, state or nation. There may be multiple Outer Settings and/or multiple levels within the Outer Setting (e.g., community, system, state).
Inner setting domain	The setting in which the innovation is implemented – a hospital, ward, unit and/or team
Individuals domain	The roles and characteristics of individuals involved in the implementation – e.g., leaders, team members, those delivering or receiving the innovation.
Implementation process domain	The activities and strategies used to implement the innovation – e.g., planning, engaging, evaluating

## Results

### The innovation domain of MCoC

The characteristics of the innovation being implemented form the starting point of a CFIR analysis [39, 40]. In this section we pay attention to the evidence for, and credibility of, the innovation (MCoC model of care delivery), its design and presentation, and its relative advantages and cost implications. We found factors related to this domain in 31 papers out of our sample of 38. Our analysis found definitional and coherence issues with the concept of midwifery continuity of carer and the related concept of continuity of care. Inconsistent terms and acronyms for the innovation were used across the literature, including ‘MCoC’ [12], ‘CoC’ [14], ‘continuity of midwife carer’ abbreviated to CMC [44] or CoMC [6], and ‘midwifery continuity of carer’ or MCoCer [45]. International third sector publications [15, 16] and the Cochrane review [10] use the term ‘midwife-led continuity-of-care’ or MLCC. In MCoC implementation research, the core innovation itself is described and defined in multiple ways, raising questions about the completeness of the body of research evidence, its reliability, transferability, and generalisability.

Using the CFIR directed us to the use of evidence for the innovation within MCoC implementation research. In general, the literature did not assess the strength of evidence for implementing MCoC. Whilst evidence for the effectiveness of midwifery continuity as an innovation was usually presented, critical analysis was often lacking, meaning that the evidential underpinning of the MCoC model was not evaluated in relation to its credibility. Most research papers, for example, stated in their opening paragraphs that the need for implementation of MCoC was derived from established advantages for women and babies. They cited as evidence a series of systematic reviews published from 2013, the most recent of which at the time of our review [10] was cited in 14 different papers. One paper cited this 2016 Cochrane review as showing MCoC ‘potentially’ improving outcomes [46], while another said it showed outcomes to be better ‘or at least as good’ as other models of care [44], but the other research presented the evidence of positive benefits for women and babies as unequivocal with limited detail (see for example [45, 47, 48]). There was no attention paid to the Cochrane review’s research object (midwife-led continuity of care) and how this might differ from the varying models of continuity of carer. Some papers also separately cited specific clinical trials [49–51] as strong evidence for MCoC effectiveness. Overall, the evidence-base in terms of benefits to women and babies, particularly clinical benefits, was routinely presented as incontrovertible, without referring to detail in the clinical evidence or the context of the MCoC implementation from which this evidence was derived.

Literature which did not cite the systematic reviews or other clinical evidence based the impetus for change on national or state level government imperatives (see for example [9, 52]), without questioning the credibility or evidence base of the policy imperatives. Only one paper engaged with difficulties in the cross-national or cross-system transferability and use of decontextualised MCoC evidence within policy implementation [11], which is a limitation in the overall body of literature.

The CFIR’s focus on identifying the persuasive aspects (referred to as ‘relative advantage’) regarding why an implementation might be initiated was also relevant. MCoC implementation was aligned with achieving core midwifery professional values [5], such as woman-centred practice [44], relational continuity as a basic principle of care [53], and working to full scope of practice [53]. A related relative advantage of implementing MCoC was the likelihood of it producing professional satisfaction for midwives [54, 55] because of its connection to ‘real midwifery’ [52, 56–59], through stronger relationships with women [57, 59–64], being at the birth [60], practicing the full scope of midwifery [19, 56, 59, 65] and practising autonomously [14, 57, 65]. In some circumstances an MCoC model could enhance recruitment of midwives [12] and result in fewer agency staff being needed [66], but these relative advantages of the innovation could be undermined by situations in which its implementation did not produce satisfactory continuity of carer outcomes. Midwives were frustrated and anxious if they were not able to provide continuity by attending the birth [55, 59, 61], and became disillusioned if they felt the model implemented damaged continuity or quality of care [58, 62], for example, if larger team sizes reduced the continuity of carer [58], or continuity including intrapartum care reduced continuity within antenatal and postnatal care [9].

Some limited attention was paid to the adaptability of the innovation in specific settings, such as team skill mix and rota arrangements [54] which are further discussed below. However, a core component of MCoC which was not systematically assessed in the literature is the centrality of on call working arrangements in different iterations of the model of care. The unpredictability of labour and birth means that if midwives are to provide continuity of carer at this point, on call duties must be an integral component of MCoC. Tensions are recognised in the literature in relation to ‘on call’ working. For some midwives, working on call is preferred to regular night shifts [56] and is preferred to shifts for some with families [59] if childcare access is easy [56], or they have a supportive partner [57]. Conversely, difficulties fitting on call working with family responsibilities [56, 57, 59, 60, 64, 67] and partner needs [57, 59] is a significant barrier to MCoC implementation. General difficulties with work life balance such as the intrusion of work into personal space [11, 57, 58], finding the work all-consuming [68, 69], being unable to switch off [55, 67], and being unable to sleep when on call [56, 64] are also potential barriers to MCoC implementation and sustainability because of on call requirements. The literature recognises that many midwives, though not all, prefer shift work to on call [11, 19, 44, 47, 70]. The non-adaptability of this core aspect of MCoC is problematic for implementation of the innovation and will be further discussed below.

Other CFIR constructs related to the MCoC innovation, such as the potential for trialling of MCoC models or issues of innovation complexity and design were not addressed in the research we surveyed. Inherent financial costs and cost effectiveness of the model were also not directly addressed in any detail.

### The outer setting domain of MCoC implementation

Analysis of the outer setting generates insights into factors involved in implementation beyond the specific locality or institution into which the innovation is introduced [39]. In the context of MCoC, this includes macro-level factors beyond the maternity service in which continuity is implemented. We found factors related to this domain in 17 papers out of our sample of 38. The availability of midwifery staff at national and regional levels impacted implementation both negatively and positively [11, 44, 71]. Recruitment of midwives is identified as a negative factor in implementing and sustaining MCoC in contexts where not enough midwives are willing to work in the model [11, 19, 12], for example due to an ageing workforce [45], or other difficulties recruiting staff because of the demands of on call working described in the innovation domain above. However, midwives who want to work in a continuity model may be attracted by the innovation [12, 66], particularly recent graduates [11, 66, 72], in settings where there is choice about whether or not to engage with MCoC [5]. Conversely, staffing may be negatively affected where MCoC models are implemented nationally or regionally without choice for staff [53]. Difficulties recruiting and retaining staff to MCoC services in different regional or rural areas have been identified in the literature [ 12, 19, 60]. The requirement to undertake on call working is also affected by macro level circumstances, such as the lack of availability of flexible and affordable childcare in the UK [11].

Factors related to formal political and policy contexts of MCoC are discussed to an extent in the literature. In the English context, a pilot trial of MCoC implementation for women at risk of preterm birth cited the national maternity policy *Better Births*, the NHS long term plan and WHO guidelines as facilitative of the implementation of MCoC [54]. In the Australian context state-level policies are sometimes explicitly described as a positive part of the implementation context (for example 52, 73) or as a potential barrier when there is a gap between government aspirations and organisational commitment [12]. Partnerships and connections with other organisations are mentioned, in relation to the midwifery curriculums of universities being helpful to implementation of MCoC in the Australian context [66, 72].

Other external policy-related factors which potentially facilitate implementation of MCoC innovation include market competition between maternity service providers for bookings [5, 19], mimetic pressure from other maternity providers [54], or performance measurement pressures compared to other service providers [44]. Pressure and opportunity for implementing change is also noted in the Outer Domain from consumer or community demand for MCoC [12, 13, 19, 44, 70]. However, the literature does not explicitly locate MCoC implementation as resulting from broader political and budgetary contexts, or ideologies and systems which promote healthcare competition. The impact of outside institutions, organisations, and structures is largely missing from the MCoC implementation literature. Informal local conditions such as social attitudes are also underexamined, with the exception of research with Aboriginal women in Australia which pays some attention to structural racism [69].

### The inner setting domain of MCoC implementation

The Inner Setting is the immediate context in which the innovation is implemented [39], in this case the maternity service, and the teams or units which implement the MCoC model. This section of the review assesses the persistent characteristics of the inner setting which affect implementation of MCoC. This was the domain with which most of the literature engaged, with findings in this domain in 35 papers out of our sample of 38. However, the inner and outer settings do not exist in isolation from each other; as has already been demonstrated, outer setting events are often influential for implementation decisions within the inner setting.

We found that the literature did not provide an analysis of pre-existing conditions within the inner setting, such as innovation capacity, existing infrastructure or workplace culture, which have been shown to affect implementation of other interventions and innovations [74]. Instead, attention focused on the unfolding implementation of the change itself, in relation to the barriers or facilitators of implementation of MCoC within the healthcare setting. As an innovation which is reliant on availability of midwifery staff, human resources being available to implement and deliver MCoC in the inner setting was a key issue in the literature. One factor we considered was whether a ‘tension for change’, defined as when a current situation is intolerable and needs to change, facilitated implementation [39] at a workforce level. Some studies identified lower levels of burnout in MCoC compared to standard midwifery work models [59, 65, 75], alongside lower levels of mental health problems such as anxiety, stress or depression [65, 75], both of which were used to argue that MCoC produces a better working environment for midwives, creating a positive tension for change at inner setting level. At the same time, other studies describe how mitigations, such as systems to support time off call, were necessary when implementing MCoC due to increased risk of burnout [19, 60], meaning that there was no tension for change based on burnout avoidance.

Institution-level solutions were found to the significant difficulties of ensuring adequate time off from the demands of on call arrangements. These included protected time ‘off call’ [56, 60, 63, 64, 67, 76] and protected annual leave [76], team work to share out weekend on call [73], and larger team size or group practice to facilitate off-call time [60, 64]. Leave coverage was found to be complex [19, 48, 58] and potentially disruptive of team-level continuity [14], with cover for unplanned or extended leave sometimes poorly organised [56, 57]. Planning and managing changes to midwives’ workload related to MCoC implementation was facilitated by local or autonomous organisation of work patterns, rosters, and caseloads [5, 11, 61, 64, 73], though additional duties such as management or specialist clinical duties were found to be difficult to manage alongside MCoC care [11, 61] and not adequately managing working time limits in some contexts could impede other team members’ time off [67]. The compatibility of workload was impeded by features of MCoC such as long hours [57] and long travel times [58, 67, 68] but could be facilitated by part-time work arrangements [11, 14, 60] and resources used to deliver the innovation such as protected time for administration [58] or clerical support [68]. Appropriate caseload sizes enabled the sustainability of MCoC [60, 62, 76], as did boundary setting by midwives in relation to women’s expectations of them [63, 67, 76].

Other inner setting factors identified in the literature relating to staffing resources included whether midwives had the skills for full scope of practice across the varying clinical contexts and demands of MCoC [14], whether skill balances and capacities within teams could be maintained [58], and whether the staffing balance between MCoC teams and hospital core midwives could be maintained [14, 19, 45]. Despite sometimes allowing for workload management, part-time work was also identified as a potential barrier to successfully implementing MCoC because rostering part-time work was hard to organise [58] and meant non part-time staff might have to do more on call [14, 61]. Succession planning [70], such as identifying new graduates who might be interested in MCoC work [72] and offering staff chances to try out working in MCoC teams [48, 57], could potentially mitigate retention issues and problems of staff not being replaced [57, 62]. Upskilling and training of midwifery staff was identified as a solution to some of these staffing issues [11, 46, 53, 66, 77].

Besides human resources, the availability of material resources in the inner setting was a factor in successful or unsuccessful MCoC implementation. Lack of space for clinical practice [44] or office functions [55, 58] was problematic, whereas provision of space was helpful to implementing the model [54, 66]. Similarly, lack of equipment or budget for equipment was problematic [52, 55, 78] and the provision of dedicated equipment and appropriate IT was helpful to implementation [46, 54, 66]. Resource availability is linked to funding, but only a limited number of papers addressed internal or external funding issues. Lack of funding for a new MCoC model was a barrier to implementation in Australia [19] and sufficient resources for the model were identified as important in Scotland and Denmark [5, 44]. Implementation itself was impeded by the lack of project management funding [19, 46] whereas a funded project manager [12, 13] or midwifery leader [54] was helpful to implementation. Specific costs related to the new way of working such as appropriate remuneration for midwives to work on call [57, 60, 62] or annualised hours [19, 46, 73], and additional costs for staff such as expenses were mentioned as potential barriers to engagement but were not examined in detail [19, 53, 55]. In relation to incentive systems, lack of accountability for delivery of MCoC within organisations could impede implementation [12] but conversely higher levels of surveillance on MCoC teams created problems for the teams who felt overly scrutinised compared to other midwifery teams [52].

### The individuals domain of MCoC implementation

This aspect of a CFIR analysis considers the roles and characteristics of individuals involved in implementing, delivering, and receiving an innovation [39]. We found factors related to this domain in 16 papers out of our sample of 38. It is notable in the MCoC implementation literature that women as service users and innovation recipients are mostly absent from the research, with the exception of a few studies where they form part of a wider study [13, 44, 54, 69]. There is published research on women’s attitudes to and experiences of MCoC, but this is not related to preparation for implementation or the ongoing delivery of the innovation. As we demonstrate further in this section and throughout the review, MCoC is a complex intervention to implement, dependent on interaction across individuals from different professional groups and services. Despite this, there is limited systematic assessment of the broad types of roles and individuals who affect implementation, with many of the research papers (*n* = 21/38) based on research with midwives only, in relation to their attitudes and experiences as implementation deliverers. In relation to other individuals involved in the implementation of MCoC, the literature finds that health service executives’ attitudes to the innovation were influential, found to be particularly problematic where they lacked motivation to implement MCoC [19, 55, 71]. Similar findings occur at the level of ‘mid-level leaders’ where other professionals including medical staff and hospital managers not understanding or valuing MCoC was a barrier to implementation [12, 13, 71] whereas support from management and midwifery leads promoted change [12, 13, 44].

Midwives’ individual attitudes to, and concerns about, delivering MCoC as the actual or potential deliverers of the innovation are covered in detail in the literature, and these connect the constructs of motivation and opportunity to deliver the innovation. The CFIR acknowledges that implementation involves subjective as well as objective assessment of barriers and facilitators [39]. Midwives were found to be concerned about personal skill levels being inadequate [68] for full scope of practice, [13, 53] and being under scrutiny [55] in terms of providing safe care [11, 44, 53] and autonomous decision making [54], especially in the context of potential litigation [53, 64] and professional visibility in the community [59]. They were concerned about impact on the institution, such as conflict with other staff [68] or destabilising the current midwifery team [13] and whether resources were available for the innovation [44]. They had concerns about personal safety such as lone working [53] or driving when tired [11] and about working patterns including on call [13, 11, 19, 44, 53, 61, 66, 68, 70] on their individual circumstance. Sometimes these concerns were represented as misguided in the literature, for example being referred to as midwives’ lack of knowledge about working in the model [13], nonetheless they must still be considered as potential barriers to MCoC implementation in the context of individual motivation, capability and need to deliver the innovation.

### The implementation domain

This aspect of a CFIR analysis looks at the activities and strategies used for implementation of an innovation [39]. We found factors related to this domain in 22 papers out of our sample of 38. Strategies found in the literature concerned with assessing the context for MCoC introduction included pre-implementation audits [54], such as skills inventory and risk assessment audits [66]. Pre-implementation engagement with others was occasionally prioritised, such as enrolling consumer groups to lobby for MCoC [12, 19], or emphasizing evidence around the outcome benefits of MCoC in a strategic manner [12, 71]. Other engagement activity was targeted at midwives as deliverers of MCoC – for example, management reassuring them about the adaptability of the new models of care [5] - or at women who might use the service, such as sessions to meet MCoC midwives [46]. Implementation was facilitated by change management [44] and the project management roles identified in the Inner Setting domain above, and by logistic planning about delivery [45]. Delivery strategies reported as helpful to implementation included gradual scaling up of the model [12, 70] or a pilot phase [54], and implementation could be hindered by allowing insufficient time [19]. Reflecting on and evaluating the implementation of MCoC or the innovation itself was a factor in some of the literature [46, 54, 58] but implementation processes connected to institutional or other structural contexts are not prominent in the literature.

Implementation researchers have noted the important influence of team capabilities, social relationships and teamwork. Much of the literature reviewed was similarly concerned with teamwork and inter/intra team relationships during the implementation of MCoC. This is connected to MCoC being concerned with human resources and working practices, as described in the innovation domain above. A key barrier to effective and sustained implementation was tension between MCoC midwife teams and other midwives such as hospital core teams who don’t work in the model but who staff the maternity wards, including confusion about roles and an ‘us and them’ team atmosphere [5, 12, 14, 19, 45, 46, 52, 54, 55, 57, 68, 69]. Tension was also described between MCoC midwives and other maternity professionals [44, 66] including staff invested in a biomedical model of maternity care [73]. Conversely, collegial relationships within midwifery teams was a facilitator of implementation and sustainability [44] as was medical and midwifery teams collaborating to implement change [54, 70], collaboration with other professionals such as health visitors [46], union involvement in negotiating change [5], and a shared vision between professionals and service users [45, 46, 54].

Team conflict and relations was the basis for tailoring strategies which facilitated implementation, such as changes to meeting arrangements to improve communications, handover, and inclusion [14, 46, 66, 73, 76]. Other tailoring strategies were aimed at fitting specific contexts such as providing training and orientation on the new model [11, 66, 72], consolidating cost centres to allow staff transfer [66], integrating MCoC into general maternity services to reduce isolated financial scrutiny [45], or in the New Zealand self-employed midwife context clarifying financial arrangements in group practices [64, 76].

## Discussion

Our thematic analysis of existing international research on MCoC implementation produced a list of factors which act as barriers to, or facilitators of, implementation of this form of care. More complex insights were gained from the subsequent mapping of these factors onto the CFIR analytic rubric. This allowed us to see absences in the literature, the balance of attention given to different aspects of implementation, and what was underexamined in the field, which are now further discussed.

### The unpredictability of birth and the implementation of on call working

The CFIR analytic process allowed us to see how the temporal uncertainty of the onset of spontaneous labour is key to the difficulties of MCoC implementation. Providing continuity that includes intrapartum care requires one midwife, or a small group of midwives, to manage birth-temporal uncertainty and adapt both their working and private lives to be present for intrapartum care for their caseload. This necessitates the implementation of on call rostering systems for midwives, resulting in individual midwives juggling scheduled antenatal care with on call intrapartum care and adaptive postnatal care, as well as managing private life demands around the unpredictable and short notice demands of work.

The MCoC innovation is, at its core, primarily concerned with deploying midwifery staff in a different way to models of care structured around delivering maternity care through specialised antenatal, intrapartum, and postpartum staffing systems. The difficulties which this engenders for midwives and maternity services considering working in this way, or trying to implement the model, are threaded throughout the implementation process, from the innovation itself, through the settings in which it is implemented, to the individuals who deliver it. The CFIR’s analytic starting point of appraising the innovation itself and its core and peripheral aspects adds a new dimension which complements existing reviews of MCoC research. For example, existing review literature has started from a position of assuming the innovation itself to be neutral or a stable “given”, finding that difficulties lie in the will of midwives to engage or “buy in” to the project [37], in the availability of workforce [28], or in the organisation and management of the systems of delivery [33, 34]. The empirical literature we reviewed, with one exception [60], did not interrogate the innovation properties of MCoC itself, nor did it critically consider its interaction with the materiality and temporality of birth and staffing requirements.

### The professional values of midwifery

The CFIR consideration of the interplay between factors within multiple outer settings and the implementation of an innovation made visible the alignment of MCoC implementation with the professional and philosophical values of midwives. Values including woman-centred care, relational care, working to full scope of practice in ‘real midwifery’, and midwife autonomy, are all important foundations of an MCoC model of care. The research reviewed shows that midwifery’s cultural alignment with MCoC facilitates implementation as it provides ‘relative advantage’ over other models of care that do not align as well with midwifery values. However, in the research we reviewed, professional midwifery values were apparent and noted, but were not critically examined. With the notable exception of research based in Denmark [5], the organisational implementation consequences of the alignment of MCoC with midwifery core values was underexamined. For example, no research examined whether professional values of midwifery had a positive (or negative) moderating influence on MCoC implementation in relation to the considerable challenges related to the necessary introduction of on call rostering.

Related to the core midwifery values which are reflected in the MCoC approach to care are the goals of midwifery as a profession, in establishing a professional area of jurisdiction [79] over which it has control, in what has been conceived as a struggle for professional recognition [4]. Burau and Overgaard demonstrate this when they describe the positive effect of the overlapping of professional and organisational goals in relation to MCoC implementation [5]. The idea that midwives should be the main or primary carers in maternity care is embedded in the very concept of MCoC, and the implementation literature repeatedly places midwives at the heart of the delivery of MCoC and its organisation, calling for midwives to be included in planning the models (see for example [45, 70]), and for their autonomy in organising and practising MCoC work to be respected (see for example [13, 14, 44]).

Questions remain over the exact impact of these core midwifery philosophies and goals on implementation, but their alignment with the values of MCoC, also visible in the policy statement of the International Confederation of Midwives [16], is likely to be a factor in the repeated, yet mostly failed, attempts to implement the innovation, for example in two major maternity reform attempts in England in the last 30 years. It is also likely to be a factor, alongside the Cochrane review [10] and research about women’s attitudes to MCoC, (see for example [80, 81]), as to why the value of the innovation itself is repeatedly and unquestionably taken for granted in the research literature. In the literature we surveyed, MCoC which embodies these core midwifery philosophies was found to be ‘the answer’ to multiple and overlapping complex maternity care delivery issues, including patient safety, quality of care, social inequalities, workforce issues, and regional healthcare provision.

### The absence of political and structural analysis

The CFIR’s attention to the outer settings of innovation was also useful in drawing our attention to the lack of contextualisation in the MCoC literature to date. In the UK context, the long history of previous and mostly unsuccessful MCoC implementation is side-lined, with some exceptions which do trace the policy back to 1993’s *Changing Childbirth* [44, 45, 53]. The political context is also not addressed, including that both the 1993 and 2016 MCoC implementation policies in the UK were adopted by Conservative governments and were led by the same person, Baroness Cumberlege. Such details may be highly relevant as the implementation of interventions such as MCoC can be directly influenced by workers’ perceptions about the source, reputation, and legitimacy of the intervention. There is also no reference to the policies of austerity (significant reductions in public spending, including healthcare) which were in place in the UK from 2010, nor to the impact of Brexit in 2020 on the healthcare workforce in the UK. There are limited references to a general lack of UK midwifery workforce [11, 44, 45], but only one paper refers to failures in workforce planning [45]. Funding, especially from outer setting agencies such as government, is underexamined in the research. When attempts to implement MCoC at scale have come about because of political agendas and policies, it is surprising to find these excluded from the analysis of implementation.

A further issue in the MCoC implementation literature is a lack of attention to indirect contextual factors in the outer setting which act as structural barriers to implementation. For example, as noted above, a barrier to midwives working on call is their difficulty in managing this alongside their non-working lives and responsibilities. Yet the literature does not discuss the mostly female midwifery workforce and the normative gendered expectations of the societies in which MCoC is implemented, such as in the UK where employed women undertake more childcare than employed men [82]. There is a lack of attention to the possible impact on implementation of poor childcare provision and availability, of transport provision and cost, or of increased housing costs and limited availability near workplaces which can all cause difficulties for NHS staff [83]. Interestingly, the implementation literature tends to invoke increased autonomy for midwives, part of the professional philosophy of midwifery, as a solution to structural and organisational barriers. For example, one literature review approvingly noted that workforce strategies making MCoC possible were ‘individually applied solutions’ within a wider maternity system [34]. Structural barriers to midwives being able to work in MCoC are only noted as problems for the individual midwife, and therefore larger scale change or adaptation which might allow implementation to happen is not considered.

### The use of evidence in MCoC implementation literature

In the course of reviewing the research, we noted several issues with the use of supporting evidence. At times the evidence cited in the literature does not effectively support the case for implementation being made. Prussing et al. [13] supports a claim in the opening paragraph that MCoC is cost effective by citing an Australian study [84], setting this reference alongside the 2016 Cochrane review as evidence for the value of MCoC. In fact, the reference concerns the development of a low-risk small MCoC birthing centre in Australia, does not focus on cost effectiveness, and cannot credibly be used to claim that MCoC is cost effective on a wide scale. In other literature, there is selective use of findings from within studies. Research which demonstrated that MCoC midwives in Australia experience less burnout, depression and anxiety than hospital midwives also showed that there was no difference in the two groups in relation to work-life balance and satisfaction with time off [65]. In citations of this research by other authors, the finding about burnout is sometimes used selectively without referencing the other finding on work-life balance [28, 53]. The small scale study is also used to support vague claims, such as that MCoC benefits midwives [34], or the concepts of burnout and work-life balance are conflated by other authors to support claims about MCoC being better than conventional models for midwives ‘in terms of job satisfaction, well-being and mental health’ [37]. Positioning citations within a paper can also give a distorted impression of the research, such as including the reduced burnout findings in the Introduction framing the paper and only including the work-life findings balance in the Discussion, so it is not necessarily clear to the reader that these are the same study [47]. Overall, the citation practices by other researchers in relation to this paper tend to amplify the findings which might support the implementation of MCoC and marginalise the findings which are neutral about the model compared to standard models.

Research which is critical of MCoC or which highlights difficulties is less frequently referred to in the overall body of literature. A 2019 study which concluded that there is an insufficiently willing workforce in the UK to implement widescale MCoC [11] is not cited by 10 of the 11 papers published subsequent to its publication. Other evidence is presented in a way which underplays differences between research studies and suggests each can be taken to add the same value to an argument, or to add value in a generalising way which exceeds the evidence base. A study of 7 midwives in one birth centre case site in Australia [85], not included in our review, travels much further than its very specific and small-scale setting. Despite the small sample size it is used by other publications in our review to evidence claims about the ideal MCoC team size [48, 72], and that midwives are content working in MCoC models [59, 76]. The study is widely used to support very strong generalised claims in other publications, such as that MCoC midwives work differently to those in non MCoC models [44], that midwives can adapt to MCoC working patterns [11], and that relations between midwives are integral to the functioning of an MCoC model [56], and that MCoC models can increase respect between midwives and other professionals [37]. The study, in Australia, is used to back up research findings from Denmark and New Zealand [59, 76]. One paper acknowledges the small scale of the study but still cites it 12 times to support different claims in their article [59]. A review paper about the job satisfaction and sustainability of working in MCoC heavily relies on this small study, using three direct midwife quotes from it [28]. It is significant to note that this use of research evidence is found in papers which are broadly favourable to MCoC as a model of care. It appears possible that biases towards *seeking out* evidence which backs up MCoC as a model are operating in these circumstances.

### Implementing MCoC – some suggestions for delivery

Despite these limitations, using the CFIR in combination with the current literature on the implementation of MCoC allows us to propose a set of implementation considerations for organisations seeking to deliver MCoC. The CFIR’s usefulness in midwifery contexts has been critiqued elsewhere as lacking in practical usefulness because of its potential to overwhelm midwives unused to change implementation [86]. Here we attempt to solve this problem by distilling our CFIR analysis of MCoC implementation literature into a list of implementation considerations for practical use in maternity contexts, presented in Table 2 below.

**Table 2:** Factors for implementers to consider when planning MCoC delivery

**Planning MCoC**
• Use dedicated and funded project managers in implementation, use logistics planning for delivery of service. • Critically evaluate the evidence on MCoC clinical outcomes and consider it in the context of the innovation inner setting. Develop a clear justification for the change and why it is relevant in this context. • Involve service users, all levels of relevant staff (e.g.midwives, managers, hospital executives, obstetricians) and as appropriate other professionals and agencies (e.g. health visitors, GPs, social services) and union representatives in MCoC implementation planning. • Consider the scope and scale of MCoC implementation and the specific services that will be offered and why (for example, will MCoC midwives attend obstetrician or screening appointments? What degree of presence is required during intrapartum care? What degree of hospital postnatal care will be provided by MCoC midwives? ). Produce clear guidelines about expectations and boundaries for service providers including MCoC and non MCoC midwives and service users. Communicate these widely. • Consider the trade-off between team size and continuity as experienced by individual women. • Plan measured introduction of the model with feedback and adjustment over time. Consider trialling the model or staged change. • Audit staffing needs and skill balances, including impact on non-MCoC teams, and whether staff workforce numbers can be met locally. Consider how core team absence cover will be managed. • Consider additional skills training needs and orientation training or mentorship systems for the new model. • Consider potential costs of the model and how these will be met, including possibilities of out-of-pocket expenses for midwives. Consider provision of dedicated equipment and space. Consider specific implementation process costs. • Consider how additional duties will be carried out under the new model and what help will be needed with administration. • Consider workflow with other systems such as hospitals or GP referrals, finance systems, relevant institutional policies. • Consider caseload levels, roster and working patterns. • Consider barriers to midwives working on call (e.g.: transport availability, transport costs, travel distances, parking, late night travel, out of hours and emergency childcare, distances in rural settings) and potential mitigations (e.g.: subsidised travel, out of hours taxis, subsidised childcare or dedicated provision, dedicated parking, overnight accommodation). Consider barriers which might rule out participation by some midwives (such as physical or mental health, disability, caring roles). • Work with local education providers to ensure a future match with organisational requirements. • Discover and address staff expectations and concerns in advance of implementation, consider the role of professional values in producing expectations and the possibility of overworking and fatigue. • Introduce new systems to service users.
**Working arrangements**
• Allow choice for midwives as to whether they work in MCoC with on call working arrangements. Allow trial periods and movement in and out of the model. • Use succession planning connected to local workforce needs. • Make detailed plans for disruption such as sickness, maternity leave, other staff absence. • Make plans for protected off call time, training time, administration time, leave arrangements, and contingency plans for unexpected disruptions. • Make plans for part time working options, consider the impact on other team members. • Discuss, consider, and manage midwife wellbeing in relation to on call, the possibility of overworking, professional isolation.
**During implementation**
• Communicate developments and solicit feedback. • Manage inter-team and inter-professional difficulties, and staff expectations and difficulties. • Develop meeting practices promoting communication and teamwork. • Monitor data in relation to care quality and safety.
**After implementation**
• Collect clear and valid data on the process and outcomes of MCoC implementation, including different types of continuity (e.g.: across whole pregnancy, intrapartum continuity) and how many different professionals women encounter. • Collect clear and valid data on the experiences and attitudes of staff and service users in relation to the innovation and ongoing implementation. • Monitor data in relation to care quality, including being curious about potential/actual impact of MCoC on patient safety, including interactions between MCoC and critical incidents, staffing levels, and clinical outcome data. • Continue succession planning and representation of the MCoC teams at executive level.

### The limitations of the CFIR

Whilst the CFIR was very useful in gaining insight into the MCoC implementation literature, we identified some limitations to its applicability and relevance. The CFIR made visible in our analysis how the materiality and temporality of labour and birth combined with the requirement of MCoC to provide continuity at the intrapartum stage (a core aspect of the innovation domain) structures the implementation of the innovation. However, the CFIR framework does not explicitly require analysis of material factors related to the innovation – in this case the unpredictability of physiological birth timing - and we suggest this would be an improvement to the framework. The innovation domain of the CFIR also does not require any analysis of the history of an innovation, focusing solely on evidence for its implementation. In the case of MCoC, this misses the fact that the innovation has been repeatedly implemented and then abandoned, for example in England, and that it has not achieved widespread use elsewhere, with the exception of New Zealand. These factors are highly relevant to assessing the innovation, but the CFIR does not pay sufficient attention to the past life of an innovation, effectively starting from the idea of innovation and then being highly future-focused. We suggest systematic examination of historic aspects of an innovation, where relevant, would be a useful addition to the framework. A further innovation domain change which would be helpful is that the ‘innovation evidence base’ construct should include not only the intrinsic robustness of evidence, but how evidence is used in persuading others about the innovation’s value.

In line with critiques already noted in the literature [39], we found that the separation between the outer and inner settings and the roles and characteristics of individuals was difficult to apply to MCoC literature. This is likely to be partly because the innovation is a staffing innovation (rather than, say, a new piece of technology) and therefore individuals and their roles are implicated all the way through the implementation. Furthermore, the separating out of individuals as a distinct domain produced a tendency to categorise difficulties they faced in implementing MCoC as their own responsibility rather than at least partly produced by structural factors in the outer or inner setting. Whilst previous critiques of the CFIR acknowledge some structural elements, they are framed as equity issues within the inner setting and expressed as normative values in implementation research [39] and we feel this does not adequately reflect the sociopolitical complexity of the barriers to implementation faced in our research. We suggest that the individuals are not separate from the multiple and overlapping settings in which innovation is implemented, and therefore this domain of the CFIR could be usefully integrated into the settings domains. We also suggest that whilst structural issues may be equity-related, they are also implementation factors in themselves and should be addressed as such.

## Conclusion

In this narrative synthesis, we have identified barriers to, and facilitators of, implementation of MCoC found in international research. Barriers to implementation included the requirement for on call working, difficulties for some midwives of integrating this working pattern with their personal lives, and difficulties in staffing the model at scale and ensuring appropriate skill mixes. Facilitators included the alignment of the innovation with professional values in midwifery, the use of local to manage on call working, dedicated resource allocation, support within the maternity service, and dedicated implementation support. Mapping these factors onto the CFIR analytic framework produced insight into previously under-discussed core aspects of the MCoC innovation, and also showed where the research literature to date is focused. There is considerable scope for future research into MCoC implementation which takes more full account of national, political, and structural context, history, and the local settings in which implementation occurs. We also identified inconsistencies in the definition of the concept of MCoC, difficulties with the use of evidence in this field, and a lack of critical engagement with core features of the innovation. More systematic and contextual evidence is needed to understand the implementation or non-implementation of midwifery continuity of carer. Existing evidence should be critically evaluated and used more cautiously in support of claims about the model of care and its implementation. However, combining the barriers and facilitators of MCoC identified in the existing literature with the CFIR framework allowed us to offer a set of factors for consideration by organisations seeking to implement MCoC. This should allow more detailed planning of implementation strategies for midwifery continuity of carer.

### Electronic supplementary material

Below is the link to the electronic supplementary material.

Supplementary Material 1. Summary table of the literature reviewed in this narrative synthesis.

Supplementary Material 2. Example of the thematic analysis process from raw data to mapping onto the CFIR.

## Data Availability

All data generated or analysed during this study are included in this published article and its supplementary information files.

## Abbreviations

CFIR: Consolidated Framework for Implementation Research
CMC/CoMC: Continuity of midwife carer
ICM: International Confederation of Midwives
MCoC: Midwifery continuity of carer
MCoCer: Midwifery continuity of carer
MeSH: Medical Subject Headings
MLCC: Midwife-led continuity-of-care
NHS: National Health Service
NPT: Normalisation Process Theory
UK: United Kingdom of Great Britain and Northern Ireland
WHO: World Health Organisation
