# Supplementary material for: Barriers and facilitators when implementing midwifery continuity of carer: a narrative analysis of the international literature
Source: BMC Pregnancy Childbirth. 2024 Aug 14;24:540. doi: 10.1186/s12884-024-06649-y (PMC11325633; doi:10.1186/s12884-024-06649-y)
Supplement: Supplementary file 1 — Supplementary Material 1. Summary table of the literature reviewed in this narrative synthesis. [file 12884_2024_6649_MOESM1_ESM.docx]

Summary table of the literature reviewed

| **Citation** | **National context** | **Setting** | **Timeframe of study** | **Model of midwifery continuity care** | **Study design** | **Focus of the study** |
| --- | --- | --- | --- | --- | --- | --- |
| Adcock et al*. (*2022) | Australia | National level | 2018 onwards | Midwifery continuity of care (MCoC), caseload and MGP | Qualitative interviews with midwifery leaders in Australia n=13 | Role of midwifery leaders in reform of maternity services towards MCoC |
| Bourgeault et al*.* (2006) | Canada | State level, Ontario | Unclear | Midwifery continuity of care, single midwife or pair of midwives | Mixed methods including qualitative interviews with  stakeholders and staff n=39 | Theoretical focus about midwives and provision of care |
| Burau and Overgaard (2015) | Denmark | 3 hospitals | 2013 | Caseload midwifery | Qualitative multiple case design including qualitative interviews with staff and managers  n=49 | Role of midwives and managers in introducing caseload midwifery |
| Callaghan et al. (2019) | UK | 1 homebirth team, urban  England | 2018 | Midwifery continuity of carer, homebirth team | Case study | Setting up of a homebirth team with MCoC |
| Collins et al. (2010) | Australia | 1 hospital | 2004 onwards | Midwifery Group Practice (MGP), teams of 6, all risk levels | Mixed methods longitudinal questionnaire of midwives n=15 | Midwives’ attitudes to introduction of MCoC |
| Cummins et al. (2018) | Australia | Multiple locations in Australia | Unclear | Midwifery continuity of care | Synthesis of 2 previous studies of qualitative interviews with graduates and managers n=13 + n=15 | Enabling new midwives to work in MCoC |
| Dawson et al. (2016) | Australia | National level | 2012 | Caseload midwifery, all models | National survey of maternity managers n=149 | Factors associated with implementation and sustainability of MCoC |
| Dawson et al. (2018) | Australia | National level | 2012 | Caseload midwifery, all models | National survey of maternity managers n=44 | Practice arrangements which sustain MCoC |
| Dixon et al. (2017) | New Zealand | National level | 2013 | Lead Maternity Carer (LMC) | National survey of midwives n=1073 | Wellbeing of NZ midwives working in MCoC |
| Farquhar et al. (1998) | UK | England, health visitors | Unclear | Team midwifery (teams of 7) | Survey of health visitors n=35 | Professionals working alongside new MCoC teams |
| Fenwick et al. (2018) | Australia | National level | 2014 | Caseload midwifery (teams of 3) | Survey of midwives n=862 | Comparison of MCoC midwife experience with non MCoC |
| Fereday and Oster (2010) | Australia | 1 hospital, urban | 2006 | Midwifery Group Practice (MGP) (buddy system) | Qualitative interviews with MCoC midwives n=17 | Midwives’ work-life balance in MCoC |
| Fernandez Turienzo et al. (2023*)* | UK | 1 hospital, preterm birth clinic, England | 2017-2018 | Caseload midwifery (buddy system) in team linked to specialist obstetrician, service for women at risk of preterm birth | Multiphase mixed method triangulation evaluation, survey n=164, interviews n=54 | Evaluation of implementation of MCoC and specialist obstetric clinic |
| Gilkison et al. (2015) | New Zealand | 1 region, rural and urban | 2011 | Lead Maternity Carer (LMC) | Qualitative interviews with MCoC midwives n=11 | How midwives sustain MCoC |
| Haines et al. (2015) | Australia | Regional Health Service, rural | 1998-2011 | Caseload midwifery group practice | Autoethnography n=3 and clinical audit | Outcomes and sustainability of MCoC |
| Hartz et al. (2012) | Australia | 1 hospital, urban | 2008-2009 | Caseload midwifery  (teams of 4) | Case study | Implementation of MCoC, facilitators and barriers |
| Jepsen et al. (2016) | Denmark | 2 hospitals | Unclear | Caseload midwifery (buddy system) | Phenomenology of practice and ethnography n=13 | Experiences of midwives in MCoC |
| Josif et al. (2014) | Australia | Remote | 2009-2010 | Midwifery group practice (team of 6), service for Aboriginal women | Mixed methods including qualitative interviews n=66 | Evaluation of MCoC for remote dwelling Aboriginal women. |
| Lewis (2020) | UK | 1 healthcare trust, unclear which nation | 2018 | Midwifery Continuity of Carer (MCoC)  (teams of 4) | Qualitative pilot service evaluation, n=5 | Midwives’ experience of MCoC re: sustainability |
| Martin et al. (2020) | UK | 1 Health Board, Scotland | Unclear | Continuity of Midwifery Care (CMC) | Survey n=321 | Baseline data on midwife attitudes to MCoC |
| McAra-Couper et al. (2014) | New Zealand | National, rural and urban | 2011-2012 | Lead Maternity Carer (LMC) | Qualitative interviews n=11 | What sustains MCoC midwives |
| McCaffery et al. (2022) | Australia | Multiple states, rural maternity service doctors | Unclear | Midwifery continuity of carer (MCoC) | Qualitative study n=10 | Doctors’ perceptions of MCoC models |
| McInnes et al. (2020) | UK | 1 Health Board, Scotland | 2016-2019 | Continuity of midwife carer (CMC)  (teams of 6-8) | Realist evaluation including survey n=321 and qualitative interviews n=13 | Implementation of MCoC |
| Menke et al. (2014) | Australia | 1 hospital, urban | 2012 | Caseload midwifery (Midwifery Group Practice), service for  socially disadvantaged women | Qualitative study (focus groups) n=17 | Midwives’ perceptions of organisational structures affecting MCoC |
| Newton et al. (2016) | Australia | State level, Victoria | 2008-2010 | Caseload midwifery | Mixed methods including surveys n=151, n=155 and qualitative interviews n=28 | Midwives’ views and experiences of MCoC and standard care |
| Newton et al. (2021) | Australia | National | 2013 | Caseload midwifery | National survey of midwives n=542 | Midwives’ views and experiences of MCoC and standard care |
| Prussing et al. (2023) | Australia | New South Wales, regional, 6 health districts | Unclear | Midwifery continuity of care models (MCC) | Qualitative interviews with midwives, managers, and women n=34 | Implementation of MCoC in regional Australia |
| Sandall (1997) | UK | 3 sites of maternity care, urban | 1994-1995 | Different models of continuity of carer | 3 comparative case sites. Interviews with midwives n=48, survey n=1166 | Impact of Changing Childbirth on sustainability of MCoC |
| Stevens & McCourt (2002)  *British Journal of Midwifery* | UK | 1 maternity service, England, urban | 1993 onwards | Caseload midwifery | Ethnography, interviews with midwives (n=12), questionnaires n=30 | Whether and how MCoC can be sustained longer term |
| Styles et al (2020)  *Women and Birth* | Australia | 1 hospital, regional, Queensland | Unclear | Midwifery continuity of care (CoC), teams of 3-4, all risk | Case study, focus groups, interviews with midwives (n= 15/16) and obstetricians (n=6/5) | Experiences of staff during implementation of MCoC |
| Taylor et al, 2019  *Midwifery* | UK | 27 maternity providers, England | 2017 | Midwifery continuity of care, any model | Survey of midwives in early adopter sites n=798 | Barriers and facilitators to working in MCoC models |
| Thorpe et al. (2022) | UK | 1 hospital, urban | 2017- 2019 | Caseload midwifery, team of 6, service for black and minority ethnic women | Case study | Setup and sustainability of an MCoC team |
| Todd et al. (1998) | UK | 1 hospital | 1995 | Team midwifery including intrapartum care, teams of 7 | Survey of midwives in one hospital setting n=80  + qualitative interviews n=22 | Midwife satisfaction with MCoC model |
| Tran et al. (2017) | Australia | Rural | 2012 | Caseload midwifery, rural birthing service | Case study using mixed methods, interviews n=9 | Insight into transition to MCoC |
| Turnbull et al. (1995) | UK | Scotland, 1 hospital, urban | 1992-1994 | Midwifery Development Unit with continuity of care, teams of 4 | Prospective cohort study, questionnaires n=85 | Attitudes of midwives to the implementation of MCoC system |
| Turner et al. (2022) | UK | NHS in general | 2018 | Midwifery Continuity of Carer (MCoCer) | Interviews with UK midwifery managers n=5 | Midwifery managers’ on implementing and sustaining MCoC in UK |
| Wakelin and Skinner (2007) | New Zealand | One health board, urban | 2006 | Lead Maternity Carer | Survey of midwives n=94 | Reasons why midwives stay or leave in the context of sustainability of LMC |
| Walker et al. (2004) | Australia | Regional health district, Queensland | Unclear | Team midwifery, team size between 5 and 8 | Focus groups with midwives n=22 | Midwives’ perceptions of team midwifery |
