# Supplementary material for: Barriers and facilitators when implementing midwifery continuity of carer: a narrative analysis of the international literature
Source: BMC Pregnancy Childbirth. 2024 Aug 14;24:540. doi: 10.1186/s12884-024-06649-y (PMC11325633; doi:10.1186/s12884-024-06649-y)
Supplement: Supplementary file 2 — Supplementary Material 2. Example of the thematic analysis process from raw data to mapping onto the CFIR. [file 12884_2024_6649_MOESM2_ESM.docx]

Additional File 2 – Example of the thematic analysis process from raw data to mapping onto the CFIR

| **Data identified in the selected literature representing facilitators or barriers to implementation of MCoC** |  | **Identification of code ideas and production of thematic connections** |  | **Mapping to the Consolidated Framework for Implementation Research** |
| --- | --- | --- | --- | --- |
| 'The need for organisations to provide the financial and human resources to support the implementation of MCoC was also identified in the data. Having a project manager was discussed...' (12)  'The importance of ‘Funding a Project Officer’ was a frequently repeated code and proposed as another major facilitator...' (13)  '...lack of interest from staff, staff shortages, and hospitals needing midwifery staff to work across both nursing and midwifery practice areas.' (19)  '...lack of medical staff support or availability...'(19)  'Funding of caseload models was identified by respondents in this study as a challenge, particularly during the establishment phase.' (19)  'Lack of consistent access to physical spaces for clinical practice.' (44)  'Midwives need a ‘base’ to work from and basic equipment...' (46)  '... the team was negatively impacted by the lack of a dedicated project manager.' (46)  '... developing a financial formula for the midwife leader post to implement the new care pathway...' (54)  ' hospitals planning to implement caseload midwifery had midwifery staff available, and there were midwives interested in working in this way' (54)  '... the organisation mandated a change by highlighting in the maternity services specification report their formal commitment to deliver high quality continuity of care for women and started organisational changes (e.g. layout change of several rooms to provide a small office space for the team, new equipment)...' (54)  'Poor resourcing left midwives feeling that they had nowhere to call home and were displaced and unsupported in their work. Midwives described being provided with out of date and ‘embar- rassing’ equipment...' (55)  '... problems with office space;...' (58)  '...other helpers such as the office managers that midwifery practices now have...' (60)  'The advent of caseload midwifery precipitated a ‘spatial’ redesign ...' (66)  '... the model has enjoyed a full complement of staff ...' (66)  'The midwives were very concerned about the absence of a dedicated physical space in the hospital to put their personal belongings, conduct appointments and do administrative tasks.' (68)  ‘...concerns about administration related to both a lack of clerical support and the amount of time involved in administration duties.' (68)  ‘…a few frustrations and delays due to a lack of a dedicated equipment budget….’ (78)  ‘…Equipment and supplies were sourced from the community budget and did not always arrive on time. [...] There was also no specific budget for communications, which impacted on our ability to promote the team.’ (78) |  | - **Material resources:** need for physical space for MCoC work, need for dedicated equipment for MCoC work - **Funding:** need for budget for MCoC implementation (project management, communication, material resources) - **Human resources:** need for adequate staffing levels and willing participants in MCoC   **All located at local maternity service level of implementation** |  | **Inner Setting Domain** Construct: J. Available Resources  **Individuals Domain, Characteristics Subdomain**  Construct B: Capability, Construct D: Motivation |
